# Contemplating the spirituality of scholarship

**DOI:** 10.1111/nup.12386

**Published:** 2022-03-21

**Authors:** David Coghlan

**Affiliations:** ^1^ Trinity Business School, Trinity College University of Dublin Dublin Ireland

**Keywords:** contemplation, interiority, nursing, scholarship, spirituality

## Abstract

Contemplation has been defined as “taking a long loving look at the real.” In the realm of the scholarship of nursing and midwifery, the pulls and counterpulls between disease and illness and between patient and person, for example, require that scholars and practitioners develop an understanding of the way their minds work and of the way they come to know. This dialogue takes a (short) loving look at the foundations of spirituality and spiritual development in human consciousness and invites readers to contemplate and appreciate their lives as scholars and practitioners.

## 1 INTRODUCTION

Contemplation has been defined as “taking a long loving look at the real” (Bartunek, 2019). Clearly anything can be contemplated. Poets and artists contemplate nature. Novelists and playwrights contemplate human nature. Parents contemplate their children. Nurses and midwives contemplate those for whom they are caring. The list is endless. This article explores contemplating the spirituality of nursing scholarship. As a first introductory note, we might clarify what we mean by scholarship. Dictionary definitions typically include elements, such as where learning is exact, scrupulous, extensive, critical, and erudite, linked to higher studies, often in a university. Over the past years, we have seen the evidence of the scholarship of those scientists who have investigated and charted the way through the virology, epidemiology, and other sciences of the COVID and of those who have constructed and administered treatments. As a second introductory note, we might clarify what we mean by spirituality. The term “spirituality” means a number of things. It may refer to a fundamental dimension of the human being, the lived experience which actualises that dimension, and the academic discipline which studies that experience. This article focuses on spirituality as a fundamental and lived dimension of the human person. We cannot understand spirituality without some personal experience of it, and hence the invitation to contemplate one's spirituality in the acts of scholarship. Is this article, distinguishing between spiritual and religious or equating them? If spirituality is understood as a fundamental and lived dimension of the human person, then it may be explicitly religious and integral to a religious tradition for some, while for others it may not.

This article takes a (short) loving look at the foundations of spirituality and spiritual development in human consciousness It begins by explores what is meant by the “real,” and what a “loving look” might entail. It locates spirituality and scholarship in the activities of the human mind and spirit, as interiority. The article as a whole is an invitation to academic readers to contemplate and appreciate their lives as scholars.

## 2 LOOKING AT THE REAL

What does it mean to say one is looking at the real? Through the influence of empiricist philosophers in the 17th century, such as David Hume, many people think of the real as what can be seen, heard, touched, tasted, and smelled, like the tree in the park. Yet, as these empiricist philosophers were unable to postulate, the real can also be intangible. A memory can be real. Anxiety or happiness can be real. Defining the real as what is out there to be seen, heard, or touched is severely limiting and may be missing much of what is “really” important in human living.

The recognisable structure of human knowing is well described by the philosopher theologian, Bernard Lonergan whose framing of the activities of human knowing have been brought to many fields: nursing theory, practice, and research (Perry, 2004), interdisciplinarity (Kane, 2013; Kane & Perry, 2016; Sawa, 2005), and health science (Daly, 2009). He describes the human knower as a subject engaging in three activities—experiencing, understanding, and judging (Lonergan, 1992). We experience some data in what we hear, see, smell, taste, and touch (what he calls data of sense) and we experience data within ourselves—our thinking, feeling, remembering, imagining, and so on (what he calls data of consciousness). We then pose questions about that experience and seek an understanding into what that experience is which test to judge if it is accurate or true. The pattern of the three activities: experience understanding and judgement is invariant in that all knowing involves experience, understanding, and judgement and applies to all settings. Within the professional fields of nursing and midwifery, an experience of a patient appearing to be unwell and subsequent questioning may reveal an insight and then to a verified judgement that it is something simple, such as a light not working, which may lead deciding that the bulb needs to be replaced. It could be that an elderly patient's vital signs are showing an abnormal reading. This could be the result of something very simple like an oximeter falling off the patient's finger or something more serious. In all these situations, the nurse is following the invariant process of human knowing of experiencing, questioning, understanding, testing that understanding, and judging that the understanding it is so or not.

## 3 LOVING

What does a taking a “loving” look at the real mean? Just as experience, understanding and judgement enables us to know the real, loving provides an added dimension. Loving takes us a step further than knowing and points to the value and worthwhileness of persons. Love provides the horizon by which we judge the value of courses of action, deciding what is good to do and becomes a motive for guiding our choices. Filde's painting, The Doctor (https://www.tate.org.uk/art/artworks/fildes-the-doctor-n01522) and the photographs of Maude Callen with her patients (https://www.dailymail.co.uk/news/article-2380359/Photos-South-Carolina-midwife-Maude-Callen-nursed-1950s-community-living-crippling-poverty.html) provide rich images of what must be a loving look.

We know that the real is not always beautiful. There is much of the real where there is evil, ugliness, disease, illness, pain, war, racism, violence, and injustice. Nurses and midwives are familiar with horrors of disease, illness, and pain. What might they contemplate beyond the horrors of a body riddled with cancer? Is it the person who is ill who is the focus of professional attention? Exploring answers to such questions opens the door to contemplation. However, to deal with such questions we need to examine further how we think and engage with different patterns of experience.

## 4 DIFFERENTIATION OF CONSCIOUSNESS

That the real as what we come to know through experience, understanding, and judgement is the first point of this consideration of contemplation. The second point to consider is how as humans beings we are polymorphic, that is we live and engage in our world through different patterns of experience—intellectual, practical, aesthetic, dramatic, and religious—and we can differentiate how we come to know in different settings, that, for example, reading a patient's chart is in a different realm of knowing to reading a novel.

Differentiation of consciousness is a notion that enables us to identify the meaning(s) in a given experience and how we are knowing in that situation. As we engage in the everyday world, we learn experientially, primarily through trial and error. This is the realm of practical knowing and where we work to complete concrete and particular everyday tasks as they arise. In the realm of the practical, we are interested in knowing, not for its own sake, but for developing effective ways of living and acting. Practical knowing operates in the everyday, descriptive language and moves fluently between action and conversation. It focuses the mind on intentions and actions, rather than the intrinsic properties of things. The practical accumulation and store of knowledge are not definitions or universally‐valid propositions but rather effective action, social rules, and norms. At its core, practical knowing describes things as they relate to us; it is a descriptive, subject‐centred context of knowing, that is not interested in universal solutions. There is also the realm of systematic and ordered explanations that are provided by theory and science. The realm of theory is not interested in things and people as they relate to us but rather they relate things to one another in a verifiable manner, such as the technical details on a patient's chart. This form of knowing operates systematically, is governed by scientific logic and uses language in a technical and explanatory manner. Explanation has to be accurate, clear, and precise so the ambiguities of practical everyday language are to be averted. Special methods are required to govern different types of investigation and hence the process of rigorous technical education and training. Theory understands things as they relate each one another. The realms of practical knowing and of theory provide different and disparate views of the world, that, for example, a patient's chart provides scientific information of the patient's medical state and sitting listening provides the practical knowledge of their emotional or psychological state. Through differentiation of consciousness, we can affirm both to be true but through different realms of knowing.

## 5 INTERIORITY

The question then is, by what mechanism do we recognise the realm of theory and the realm of practical knowing and be able to move from one realm to another, appreciating the value of both without confusing them. The third realm, interiority, emerges as the answer to this question. Interiority is the process whereby we catch ourselves in the act of knowing by attending to how we are knowing when we are in the realm of practical knowing or the realm of theory (Coghlan, 2010). This is a personal process in which we heighten our awareness of ourselves, as we undertake activities such as knowing and doing and bring them into our conscious awareness. Thus, we can discover that our knowing process operates at four levels: the empirical level of our experiencing, the intelligent level of our understanding, the rational level of our reflection, marshalling evidence and judging, and the responsible level of our decision‐making and acting.

The distinction between practical, theory, and interiority as realms of meaning calls for a heightened consciousness so that we can move from the outer world of practicality and theory/science to the inner world of ourselves as knowers. Interiority involves moving from *what* we know to *how* we know. Interiority involves using our knowledge of how we know to critique what realm of knowing is appropriate for a given situation. For instance, there is time for a nurse to engage in a diagnostic study of a patient's chart and a time for conversing with the patient. The infrastructure for interiority is characterised by the conscious operations of: attending what gives us curiosity, delight, anxiety, and so on; by adverting to what is it we do not yet understand, the dissatisfaction with current explanations, the puzzled search for new understanding, the release when we receive insights, and our efforts to express what it is that we have understood; by attending to our reasonableness, whether our understanding is coherent or true, whether something will work or not and by attending to the responsibilities of our action. Interiority enables the mediation between the pulls and counterpulls of disease and illness, of research and teaching, and of patient and person. It is grounded, not in any thesis or grand theory, but in the recognisable and verifiable operations of human enquiry as we experience, receive insights, come to understand our understanding, and valuing and try to be faithful to them

Interiority, then, is at the heart of contemplation—that in our looking at the real, we can attend to the data of our consciousness and attend to differentiating theoretical/scientific, practical, and aesthetic patterns of knowing. Contemplation is not merely a physical staring, an activity that Kahneman (2011) calls WHYSIATI (what you see is all there is). As Florence Nightingale stated “merely looking at the sick is not observing…to look is not always to see” (Holton, 1984, p. 64). Rather it is a looking that sees, that appreciates the beauty in a craft, in a scenery, in the flow of language, in smiles, in acts of kindness, in friendship, in an idea…. Contemplation or taking a long loving look at the real may be through mindfulness, painting, photography, prayer, worship, poetry, reading, medical care, and providing nursing and midwifery care. Contemplation is a whole person activity as it is a whole person that we encounter the real.

## 6 SPIRITUALITY

This article is presenting the foundations of spirituality in human consciousness (O'Sullivan, 2019). The point underpinning the discussion of differentiation of consciousness is that spiritual integration does not take place within one realm of meaning alone. It allows us to integrate the realms of practical knowing with that of theory and these realms with the realm of religious transcendence, art, and scholarship. Spiritual integration is the capacity to move through the different reals of meaning intelligently and be able to move from one to another as a situation demands. The spiritually integrated person overcomes the divisions of the split soul because they understand and distinguish the aims and methods of both practical knowing from theoretical analysis and can ground their lives in working of their minds and their efforts of live responsible lives. Spiritual integration is a commitment to using the head and the heart, not only about the outer world but about the head and the heart themselves. Spirituality is not merely a body of knowledge but a habit of the soul. This regards the data of consciousness as the testing ground for any theory about how we use our capacity for contemplation. It is by attending to our interiority, we can appreciate the spirituality that is latent in human cognition as we go beyond practical knowing and theory and appreciate the eros of the human mind in our knowing, acting and desiring (Snedden, 2017).

## 7 SCHOLARSHIP

When we discuss scholarship, we are engaging in the world mediated by meaning. An individual may ask herself, Is my life of scholarship a meaningful way of living? How is it meaningful? While in many disciplines scholarship has got drawn into a war of measurement impact and ranked journals, this is a selected part of our lives as scholars. In the context of university‐based scholarship, scholarship seems to have three areas: pursuing areas and questions of interest, shaping the minds of students and future scholars, and making some contribution to the running and development of our respective schools and departments. At different times of our working lives, these may have different emphases and importance. They make different demands (both welcome and unwelcome) on time, energy, and motivation and on how we might look long and loving at them.

How does the differentiated consciousness, especially interiority, inform the process of being a scholar? By focusing on how we come to know, which begins from experiencing and posing questions to those experiences (Coghlan, 2020). The answers or understandings that come have to be subjected to rigorous scrutiny in light of how they fit the evidence and whether there are alternative explanations. The outcome is a judgement that it is indeed so and the model/theory is affirmed. If not, the process of experiencing, inquiring, and testing continues.

How then might we contemplate a spirituality of being a scholar? In their exploration of the process of theorising (which is as important as focusing on theory as an outcome), Hansen and Madsen (2019) show how it involves attending not only to external data but also to the internal data of one's own thinking and assumptions and engaging in a community through reading, talking, listening, questioning, and writing. In short, theorising places the emphasis on the scholar in scholarship. This article is proposing that it is by attending to our interiority and appreciating how we come to know, rather than looking externally for models (though, i.e., useful too) that we can a) discover the core of our scholarship and b) take a long loving look at it.

## 8 CONCLUDING REMARKS

I have presented the notion of contemplation as taking a long loving look at the real. I've challenged the notion of the real as being what one sees is all there is and argued for the real as what is living and pulsating. I've suggested that looking is not staring. Therefore, our looking needs not to be hurried, involving what Kahneman refers to as slow thinking, and that our looking be wondering, loving, appreciative and compassionate, especially when the immediate is ugly and painful. Earlier I cited Snedden as arguing that it is by attending to our interiority we can appreciate the spirituality that is latent in human cognition as we go beyond practical knowing and theory and appreciate the eros of the human mind in our knowing, acting, and desiring as scholars.

The scholar is first of all a person—there is a scholar in scholarship. The poet, Mary Oliver, writes that that at the end of her life, she would like to be able to say that she had lived rather than to have visited (http://www.phys.unm.edu/%7Etw/fas/yits/archive/oliver_whendeathcomes.html). Some scholarly work is visiting, perhaps in the superficial gathering of data or when making technical interventions. Contemplation draws us to live, not merely to visit.

## CONFLICT OF INTEREST

The author declares no conflict of interest.

## Data Availability

As this is a reflective dialogue paper, there are no data.

## References

[nup12386-bib-0001] Bartunek, J. M. (2019). Contemplation and organization studies: Why contemplative activities are so crucial for our academic lives. Organization Studies, 40, 1463–1479.

[nup12386-bib-0002] Coghlan, D. (2010). Interiority as the cutting edge between theory and practice: A first person perspective. International Journal of Action Research6, 6(2–3), 288–307.

[nup12386-bib-0003] Coghlan, D. (2020). Developing a spirituality of scholarship: A first person methodological approach. Studies in Spirituality, 30, 33–46.

[nup12386-bib-0004] Daly, P. R. (2009). A theory of health science and the healing arts based on the philosophy of Bernard Lonergan. Theoretical Medical Bioethics, 30, 147–160. 10.1007/s11017-009-9101-9 19283513

[nup12386-bib-0014] Hansen, A. V. , & Madsen, S . (2019). Theorizing in organization studies: Lessons from key thinkers. Edward Elgar.

[nup12386-bib-0005] Holton, S. (1984). Feminine authority and social order: Florence Nightingale's conception of nursing and health care. Social Analysis: The International Journal of Anthropology, 15, 59–72.

[nup12386-bib-0006] Kahneman, D. (2011). Thinking, fast and slow. Penguin Press.

[nup12386-bib-0007] Kane, A. T. (2013). Lonergan's philosophy as grounding cross‐disciplinary research. Nursing Philosophy, 15, 125–137. 10.1111/nup.12024 24741692

[nup12386-bib-0008] Kane, A. T. , & Perry, D. J. (2016). What we're trying to solve: The back and forth of engaged interdisciplinary inquiry. Nursing Inquiry, 23, 327–337. 27550841 10.1111/nin.12147

[nup12386-bib-0009] Lonergan, B. J. (1992). The collected works of Bernard Lonergan, insight: An essay in human understanding (Vol. 4). Toronto University Press.

[nup12386-bib-0010] O'Sullivan, M. (2019). Authentic subjectivity as a methodology for studying spirituality. Mysterion, 12(2), 271–278.

[nup12386-bib-0011] Perry, D. J. (2004). Self‐transcendence: Lonergan's key to integration of nursing theory, research and practice. Nursing Philosophy, 5, 67–74. 15043699 10.1111/j.1466-769X.2004.00166.x

[nup12386-bib-0012] Sawa, R. W. (2005). Foundations of interdisciplinarity: A Lonergan perspective. Medicine, Health Care, and Philosophy, 8, 53–61. 15906939 10.1007/s11019-004-2422-6

[nup12386-bib-0013] Snedden, E. J. (2017). The eros of the human spirit. Paulist Press.

